# RET in breast cancer: pathogenic implications and mechanisms of drug resistance

**DOI:** 10.20517/cdr.2019.66

**Published:** 2019-12-19

**Authors:** Cristiana Lo Nigro, Marta Rusmini, Isabella Ceccherini

**Affiliations:** ^1^Laboratory Department, S. Croce & Carle Teaching Hospital, Cuneo 12100, Italy.; ^2^U.O.C. Medical Genetics, IRCCS Istituto Giannina Gaslini, Genoa 16147, Italy.

**Keywords:** Breast cancer, RET, hormone resistance, receptor tyrosine kinases inhibitors

## Abstract

Initiation, progression, outcome and sensibility to therapies in breast cancer (BC), the most frequent cancer in women, are driven by somatic and germline mutations. Although the effectiveness of hormonal therapies is well-founded, it is prescribed for cancers which express steroid hormone receptors, such as estrogen receptor (ER). *RET* is a proto-oncogene encoding a transmembrane tyrosine kinase receptor that is activated by one of its four ligands (GDNF, neurturin, artemin or persephin) and one of its coreceptors (Gfrα1-4). Loss-of-function mutations in RET are responsible for Hirschsprung disease, while gain-of-function mutations for multiple endocrine neoplasia type 2. In addition, deregulation of its intracellular signaling, due to mutations, gene rearrangements, overexpression or transcriptional upregulation, can cause several neuroendocrine and epithelial tumors. In BC, amplification of receptor tyrosine kinases, such as ERBB2, EGFR, IGFR and FGFR1, and/or their upregulation contribute to cancer initiation and progression. RET can also have an important role in BC, but only in the subset of ER-positive (ER+) tumors, where it is found overexpressed. Targeting the RET pathway and shedding light on molecular basis of the resistance to hormone therapy may lead to new therapies in ER+ BC, improving treatment outcome and preventing tumor-related events. Thus, here, we review the state of the art of RET biology in BC and agents targeting RET tested in the clinical trials and discuss the specificity of the still available RET inhibitors and the molecular mechanisms underlying the BC resistance to endocrine therapy.

## Introduction

Breast cancer (BC) is the most common cancer among women, with approximately 1,700,000 new cases each year and a median survival in the metastatic setting of ~24 months, thus representing a major worldwide health problem^[1-3]^. Similar to other cancers, genetic causes as well as cellular and environmental factors play roles in BC onset and progression. Germline and somatic mutations of genes involved in inherited cancer syndromes^[4,5]^, such as BC gene 1 (*BRCA1*) and BC gene 2 (*BRCA2*), and/or related with specific morphological stages^[6]^ and response to therapy^[7-9]^ may influence the BC risk and its outcome. The availability of a number of distinguishing features to classify different subtypes of tumors allows stratifying patients for the most appropriate treatments. In particular, several subtypes of BC can be recognized on the basis of expression of estrogen receptor-alpha (ERα), human epidermal growth factor receptor 2 (HER2) and progesterone receptor (PR)^[10]^. Different patterns of gene expression characterize the biology underneath each subtype^[11]^, thus accounting for distinct clinical responses in terms of treatment outcome and pattern of recurrence and survival^[12-14]^.

Despite improved screening and treatments and rising survival rates, BC is still the most invasive cancer in women. Standard therapy combines chemotherapy with targeted drugs and a hormonal approach, with only women affected with tumors expressing steroid hormone receptors (70% of total cases) having access to this latter treatment^[15-17]^. The endocrine therapy aims at limiting the growth and survival of ER-positive (ER+) cancers^[18]^ by means of the promotion of ER degradation using specific ER downmodulators (e.g., fulvestrant), the antagonized binding of estrogens with selective ER modulators (SERMs) (e.g., tamoxifen), or blocking estrogen synthesis by aromatase inhibitors (AIs)^[19]^, which are also the elective therapy for post-menopausal women with ER+ BC^[20]^.

However, the success of the therapeutic strategy is often limited by acquired or *de novo* resistance^[21-23]^, as in the case of AI, for which several molecular pathways seem to be involved in the resistance developed by patients^[18]^.

Understanding how ER+ BC metastasizes is critical since the major cause of death in BC is metastasis to distant organs. Results from many studies suggest dysregulation of the estrogen receptor alpha gene (*ESR1*) contributes to therapeutic resistance and metastatic biology^[24]^. Lei *et al*.^[24]^’s review covers both pre-clinical and clinical evidence on the spectrum of *ESR1* alterations including amplification, point mutations, and genomic rearrangement events driving treatment resistance and metastatic potential of ER+ BC. Importantly, we describe how these *ESR1* alterations may provide therapeutic opportunities to improve outcomes in patients with lethal, metastatic BC^[24]^.

Indeed, cancer cells can either become hypersensitive to residual estradiol (E2), remaining dependent on ER signaling for their proliferation^[25]^, or possibly elude the inhibitory action of AIs by activating E2 in a ER-independent way. In any case, epidermal growth factor receptor (EGFR), HER2 or insulin-like growth factor receptor (IGF-IR) overexpression^[26,27]^ would lead to the activation of the MAPK and PI3K/AKT signaling cascades that induce ER phosphorylation, cell growth and survival^[28]^. Therefore, the combination of AIs with therapies targeting ER-related pathways could be effective in both enhancing AI therapy response and preventing resistance.

However, almost all ER+ BC patients develop resistance to ER-directed agents in the metastatic setting. Apart from mutations in *ESR1*, which occur in 25%-30% of BCs treated with AI, knowledge about resistance mechanisms remains incomplete. In the BCs studied by Nayar *et al*.^[29]^ (2019), *ERBB2* and *ESR1* mutations are mutually exclusive, suggesting a distinct mechanism of acquired resistance to ER-targeted drugs. *In vitro* analysis confirmed that the *ERBB2* mutations conferred estrogen independence and, differently from *ESR1* mutations, also resistance to tamoxifen, fulvestrant and the cyclin dependent kinases CDK4 and CDK6 inhibitors. Resistance was overcome by combining ER-directed drugs with HER2 kinase inhibitors^[29]^.

Moreover, resistance to hormone therapy has also been studied and mainly accounted for by the signaling talk between growth factor receptor tyrosine kinases (RTKs) and ER^[30]^.

RTKs are known to play a role in cancer development, their mutations deregulating many biological processes that are under their control, particularly once constitutively activated or when their signaling pathways are altered^[31]^.

For this reason, therapies aimed at counteracting the effect of RTKs activation have already been adopted in different kinds of tumors such as non-small cell lung cancer (EGFR and gefitinib), gastrointestinal stromal tumors (c-KIT and gleevec), and BC (HER2 and herceptin)^[32]^.

One of the RTKs playing a central role in BC and, in particular, in the ER+ subtype, is RET (REarranged during Transfection)^[33]^.

RET activation by the binding of one of its four soluble ligands [Glial cell Derived Neurotrophic Factor (GDNF), Neurturin (NRTN), Artemin (ARTN) or Persephin (PSPN)] and one of four GPI-linked coreceptors (GFRα1-4) leads to its dimerization and autophosphorylation of tyrosines in the intracellular tyrosine kinase domain^[34,35]^. Germline mutations are responsible for two different disorders depending on whether they induce loss-of-function, as in Hirschsprung’s disease (HSCR), or gain-of-function, as in Multiple Endocrine Neoplasia type 2 (MEN2). Consistent with the dominant occurrence of the RET-related tumors, somatic mutations are found in sporadic Medullary Thyroid Carcinoma (MTC). Gene rearrangements are also possible and they have been found in Papillary Thyroid Carcinoma (PTC) and, recently, in lung adenocarcinoma^[36]^ [Table 1].

**Table 1:** RET-altered cancers and percent of RET alterations

RET-altered cancers	RET point mutation	RET rearrangement	Ref.
MEN2			
MEN2A	98%-100% (codon 634 in 85% of cases)		[37-40]
FMTC	88%-98%		
MEN2B	M918T (95%), A833F (5%)		
Sporadic MTC	43%-71%		[41-44]
Papillary thyroid carcinoma (PTC)		10%-40%	[45,46]
Non-small cell lung cancer (NSCLC)		1%-2%	[47,48]
Others (chronic myelomonocytic leukemia and colorectal, ovarian and head and neck tumors)		1%-2%	[49-51]
Intraductal carcinoma		40%	[52]
Spitzoid tumors		3%	[53]
Breast cancer		0.2%-1.2%	[48,54]

To further understand the genetic causes of RET-related diseases, and especially its role in the complex inheritance of some of them, common *RET* SNPs have also been studied^[55-58]^. In particular, the so-called *RET*+3 SNP (rs2435357), affecting an intronic enhancer and thus reducing *RET* expression, presents a predisposing genetic factor for HSCR disease, while it is underrepresented in sporadic MTC^[58,59]^.

The involvement of *RET* in the pathogenesis of BC has been confirmed by several independent studies^[51,60]^. A subset of ER+ tumors showed an overexpression of *RET* and *GFRα1*^[61-63]^, which correlates with decreased metastasis-free survival^[64,65]^, thus confirming the importance of RET in the development of ER+ cancers and making it a promising target to avoid tamoxifen resistance and improve effects of hormone therapy in BC^[60]^.

In this review, we emphasize the *RET*-mediated pathogenic mechanisms leading to BC and provide the state of the art on RET targeted inhibitors as therapeutic drugs, combined to overcome drug resistance in selected BC patients.

## RET related mechanisms and RET inhibitors underlying resistance to antitumor therapy in BC

### RET-ER crosstalk in BC

The proto-oncogene *RET* is located on chromosome 10 (10q11.2) and is composed of 21 exons. The encoded receptor tyrosine kinase consists of three regions: the extracellular, the transmembrane and the intracellular portions. The N-terminal extracellular region is composed of a cadherin-like domain that guarantees conformational changes necessary for its interaction with ligands and coreceptors and a cystein-rich domain, which is important for the tertiary structure of RET through the establishment of intramolecular disulfide bonds. The intracellular portion of the receptor contains two tyrosine kinase domains involved in several intracellular signaling cascades regulating cell proliferation, differentiation, chemotaxis and migration. Consistent with its main role in the enteric neuronal system development^[35,66]^, *RET* is expressed by neural crest-derived cells.

Apart from the peripheral enteric, sympathetic, and sensory neurons, thymus and testis, a very low amount of the RET protein is expressed in adult tissues^[67]^.

**Figure 1:**
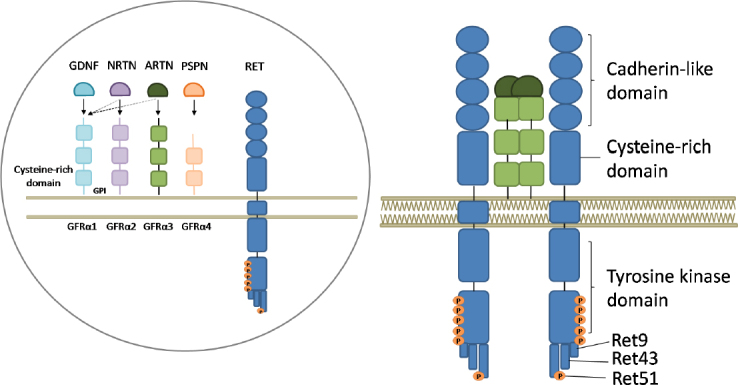
Representation of the RET receptor, its ligands and coreceptors and its activation mechanism. Inside the circle: RET is represented together with the soluble ligands glial derived neurotrophic factor (GDNF), neurturin (NRTN), artemin (ARTN) and persephin (PSPN) and the coreceptors GFRα1-4 anchored to the membrane by glycosylphosphatidylinositol (GPI) domains. The full arrows indicate the principal interactions, while the broken ones indicate possible interactions. Outside the circle: activation of RET. Upon ligand-coreceptor interaction, the complex binds RET, leading to its dimerization and activation through tyrosine auto-phosphorylation. Six tyrosine residues in the intracellular part of the receptor, involved in its activation, are represented by orange dots and the three principal isoforms of RET are indicated as RET9, RET43, and RET51

The *RET* activation process starts with one of the four ligands belonging to the Glial cell-line-derived neurotrophic factor (GDNF) family (GDNF, Neurturin, Artemin or Persephin) binding one of the glycosylphosphatidylinositol membrane anchored coreceptors GFRα1-4 and RET itself. The three-protein complex is then recruited to the lipid rafts and RET molecules dimerize, thus inducing the autophosphorylation of tyrosines in the intracellular domain^[68,69]^ [Figure 1].

RET exists in two different isoforms obtained by different splicing of the distal exon: RET9 and RET51 differ for their C-terminus length (9 and 51 amino acids, respectively). Despite the limited knowledge about the function of the 2 *RET* isoforms, RET51 seems to be more important in tumor development^[70,71]^. A third isoform has been identified and named RET43 as the result of the replacement of exon 20 with exon 21^[72]^.

Germline gain-of-function mutations in *RET* cause the MEN2 syndromes^[35,73]^, including MEN2A, MEN2B, and Familial MTC (FMTC). In particular, MEN2A is characterized by MTC, pheochromocytoma (a tumor of the adrenal chromaffin cells) and hyperparathyroidism (HPT). Rarely it also presents with amyloidosis or HSCR. MEN2B associates with a severe form of MTC, pheochromocytoma, mucosal ganglioneuromatosis, and marfanoid habitus^[74-76]^. Ninety-five percent of MEN2B cases are due to a mutation at codon 918 of the *RET* gene (M918T), in the tyrosine kinase 2 domain. A small percentage (5%) of cases are caused instead by mutations at codon 883 (A883F). MTC is therefore a tumor shared by both syndromes and arises from the thyroid C-cell, secreting calcitonin and derived from neural crests.

**Figure 2:**
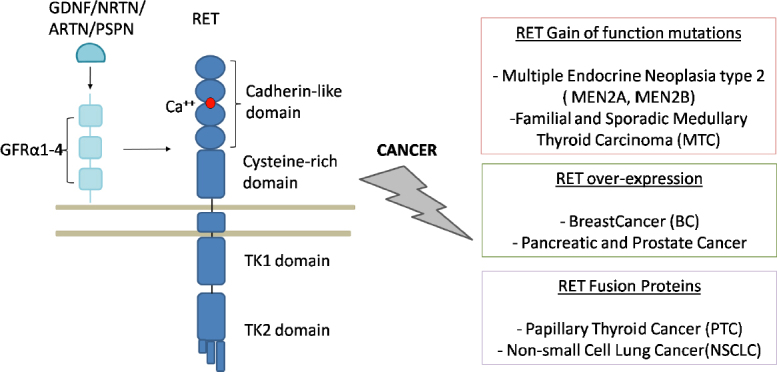
RET and RET-related cancers (adapted from Morandi *et al.*^[60]^, 2011). On the left: physiological activation of RET receptor tyrosine kinase with the representation of the molecules interacting in the formation of the complex that activates the intracellular cascade. All the RET domains, namely cadherin-like, cystein-rich, transmembrane and tyrosine kinases domains, are represented together with the calcium binding site. On the right: *RET* involvement in cancers. Germline and somatic point mutations cause MEN2 and MTC, respectively; the overexpression of the receptor has been observed in breast cancer, both ERα and endocrine resistant, and in prostate and pancreatic cancers; the fusion between the intracellular part of RET and the amino-terminal of different proteins cause PTC and NSCLC

Conversely, PTC derives from thyroid follicles as the result of DNA breaks involving *RET* and another unrelated gene: the successive balanced translocation of the 2 broken ends leads to the fusion of the C-terminus of *RET* to the N-terminus of the other gene, thus inducing *RET* to be constitutively expressed^[77]^. Indeed, the N-terminal portion of the RET chimeric proteins physiologically dimerizes and RET tyrosines are auto-phosphorylated. The breakpoint of *RET* occurs, almost exclusively, in intron 11 and produces proteins missing the transmembrane domain. These constitutively active cytoplasmic chimeric proteins are named RET/PTC^[78,79]^ [Figure 2].

Many pieces of evidence highlight the importance of RET in ER+ BCs. *RET*, in fact, has been identified as a novel gene, upregulated by ER in BC specimens^[61]^ and overexpressed together with its coreceptor GFR±1 in a subset of ER+ tumors^[62]^. Studying two ER+ cell lines, MCF7 and T47D, Boulay *et al*.^[63]^ (2008) showed that GDNF stimulation activated signaling pathways that increase, in a RET-dependent manner, anchorage-independent proliferation.

Moreover, by analyzing two independent BC tissue microarrays, Plaza-Menacho *et al*.^[64]^ (2010) found that *RET* expression was associated with ER+ tumors and that there was a 2-fold increase in *RET* positive samples from patients who subsequently developed tamoxifen resistance compared to non-selected invasive BC^[64]^.

From the analysis of the TCGA dataset, a total of 394 somatic mutations of the *RET* gene, namely 373 single base substitution, 10 small deletion, and 11 small insertion, has been detected in 339 samples from a variety of different tumors. If the search is restricted to BC, nine somatic *RET* single nucleotide variants and two somatic *RET* indels have been found in 104 tumor samples (see http://www.proteinatlas.org).

Expression data of the *RET* gene in a variety of tumors are available at TCGA^[80]^; in particular, BC results to be, among other tumors, the one with highest *RET* expression (for raw and elaborated data with graphs and figures, see https://www.proteinatlas.org/ENSG00000165731-RET/pathology).

The evidence that *RET* is more expressed in ER+ than in ER- cancers is tightly correlated to the involvement of ER pathway in the development and progression of BC^[81]^. In particular, along with the co-expression of *RET* with ER-linked genes in BC cell lines and primary cancers, an increased activation of its promoter has been demonstrated with ER stimulation by estrogen. Moreover, ER regulates *RET* gene transcription through multiple estrogen response elements binding sites present in its promoter region^[82,83]^.

Given the identification of *TFAP2C* as regulator of ER-associated genes, Spanheimer *et al*.^[84]^ (2013) defined 5 main binding elements for TFAP2C in the *RET* promoter through ChIP-seq experiments^[83-86]^. TFAP2C belongs to the AP-2 family of transcription factor modulators with a GCCNNNGGC consensus sequence^[87,88]^.

The role of TFAP2C as regulator in BC has been postulated based on its level in luminal BC and its regulation of ER and therefore, both directly and indirectly, of several genes in the ER-associated expression cluster^[84]^. Moreover, TFAP2C expression in BC is linked to shortened survival and hormone resistance (HR), which is, at least in part, due to regulation of *RET*.

Since *RET* is also expressed in subsets of ER- tumors, the role of TFAP2C in regulating *RET* expression was also investigated in ER- BC cells. In particular, the ER- cells MDA-MB-453 were taken into consideration and *RET* was consistently found to not respond to estrogen treatment^[84]^. In particular, it was found that TFAP2C is able to induce both ER-independent *RET* expression and hormone responsive mechanisms, thus suggesting that different sets of coactivators could compete in different phenotypes of BC.

Spanheimer *et al*.^[81,84]^ showed that RET and ER± regulate cell proliferation through distinct pathways in luminal BC, defining the functional role of RET-ER interactions and the potential of combined therapy targeting these two pathways^[81,84]^. In fact, as TFAP2C controls both ER and *RET*, the knockdown of this gene leads to a greater effect on cell growth than either *RET* or ER alone. Nevertheless, tamoxifen and sunitinib have confirmed enhanced effectiveness of the ER and RET pathways inhibition in regulating cell growth.

A similar approach was used to investigate the regulation and role of EGFR in luminal BC^[89]^ where the knockdown of TFAP2C has induced decreased expression of EGFR in a panel of this tumor and, consistently, the *EGFR* gene has resulted by ChIP-seq to be a TFAP2C target.

In MCF-7 cells, the treatment with the TKI vandetanib was effective on tumor growth; this response is eliminated by dual knockdown of *RET* and *EGFR*, thus establishing a link between expression of *RET/EGFR* and response to TKIs.

In conclusion, TFAP2C modulates EGFR in luminal BC and its targets EGFR and RET have been shown to mediate the response to vandetanib.

### Anti-RET drugs

BC has been associated with activating mutations in tyrosine kinases and, in particular, genetic alterations of the *RET* gene, including germline and somatic mutations, overexpression, amplifications and rearrangements^[90]^.

In oncology, the tyrosine kinase family represents a significant druggable target and, consistently, effective drugs for cancer therapy have been identified^[79]^, such as several small molecules targeting the kinase nucleotide-binding pocket and thus blocking the phosphorylation activity.

Imatinib is the first clinical Tyrosine Kinase Inhibitor (TKI), which was approved in the early 2000s. Despite its likely effect on RET^[76]^, it poorly performed in MTC patients^[91]^ when compared to its primary targets ABL, platelet-derived growth factor receptor (PDGFR) and KIT.

In the following paragraph, we report the results of clinical trials involving *RET* related cancer patients while for preclinical data another review can be accessed^[75]^.

Despite the big effort to find selective RET kinase inhibitors, only multikinase inhibitors with a significant activity against RET could be identified up to now^[92]^.

Originally, vandetanib (ZD6474) was developed as a second generation of EGFR TKI, but its unexpected anti-neoplastic activity with a great specificity for RET receptor^[93-95]^ elected its use in the treatment of metastatic MTC. Vandetanib-treated murine models with high RET or EGFR have regressed in HER2 and triple negative (TNBC) tumors, where *RET* expression resulted to be high^[69]^. This effect seems to be related both to a significant decrease in RET or EGFR phosphorylation and to MAPKs inhibition. Moreover, this drug has been shown to be an inhibitor of RET activated focal adhesion kinase (FAK) phosphorylation^[96,97]^ that is more potent than phospho-RET and phospho-ERK^[81,98]^. In 2018, Li *et al*.^[99]^ suggested vandetanib to also be a potent inhibitor of cell proliferation, by regulating cell cycle and apoptosis. Vandetanib-treated tumors showed a decrease at both mRNA and protein level of mechanistic targets of rapamycin (mTOR), hypoxia-inducible factor-1 (HIF-1) alpha, and vascular endothelial growth factor (VEGF), genes allowing survival, proliferation and tumor growth and all up-regulated in BC. This results in the inhibition of wound healing, invasion and tubular formation *in vitro* and *in vivo*^[100,101]^.

Another potent inhibitor of RET enzymatic activity is sorafenib, which has comparable efficiency at nanomolar concentration *in vitro* and acts on both RET wt and RET V804M. Moreover, *in vivo*, it inhibits RET phosphorylation, downstream signaling and cell proliferation^[29,102,103]^.

Sunitinib is a non-selective TKI with anti-RET activity, which also acts against a number of RTKs, such as VEGF receptor (VEGFR1-3), PDGFR-±, PDGFR-β and KIT^[10,81,104,105]^. However, its expression and role in BC need to be further investigated.

The Food and Drug Administration has approved the use of cabozantinib, TKI showing potential anti-RET activity by an unknown mechanism^[106-108]^.

Both sunitinib and cabozantinib, together with inhibitory small molecules NVP-BBT594 and NVP-AST487, were investigated and checked in combination with the AI letrozole in ER±+ BC^[109]^. NVP-AST487 acted as the best inhibitor abrogating the GDNF-RET pathway and the growth of 3D tumor spheroids.

The RET antibody Y078 was linked to the DM1 and DM4 derivatives of the strong microtubule-targeted compound, the cytotoxic maytansine, to generate Y078-DM1 and Y078-DM4^[67]^. The cytotoxicity and activity of these compounds were both tested in human BC cell lines. Moreover, cytotoxic activity, dose-dependent, reversible alterations in blood chemistry, and development of on-target neuropathy were demonstrated upon a single-dose of Y078-DM1 in cynomolgus monkeys.

As thyroid and lung cancers are often generated by fusion proteins involving RET, Paratala *et al*.^[54]^ (2018) functionally characterized two RET fusions, named NCOA4-RET and RASGEF1A-RET, which showed oncogenic activity due to the activation of RET kinase, MAPK and PI3K pathways. This explains the case of metastatic BC progressing on HER2-targeted therapy where the NCOA4-RET fusion was identified.

Thus, the mechanism that could explain the oncogenic activity of fusion proteins of RET might be related to RET capability of dimerization and activation in a ligand-independent manner, thus resulting in increased cell survival and proliferation.

More recently, novel selective RET inhibitors (BLU-667, LOXO-292 and RXDX-105) have been investigated in early phase clinical trials in NSCLC, showing promising efficacy with a manageable toxicity profile^[110]^. In particular, BLU-667: (1) demonstrated increased efficacy over approved MKIs against oncogenic RET variants *in vitro*; (2) inhibited growth of NSCLC and thyroid cancer xenografts; and (3) in first testing in patients with RET-altered NSCLC and MTC, significantly inhibited RET pathways and induced durable clinical responses without notable off-target toxicity^[111]^. The Phase I/II LIBRETTO-001 basket trial (NCT03157128) is investigating the safety, tolerability, pharmacokinetics and preliminary antitumor activity of LOXO-292 in patients with RET rearranged solid tumors. The first results of RET-driven NSCLC patients were reported at the American Society of Clinical Oncology Annual 2018 meeting and updated at the 19th IASLC World Conference of Lung Cancer^[110]^. Data reported from this trial at the European Society For Medical Oncology Annual Meeting 2019 formed the basis for the US Food and Drug Administration (FDA) breakthrough designation that was granted for LOXO-292 in the treatment of RET fusion-positive NSCLC, RET fusion-positive thyroid cancer, and RET-mutant MTC. In parallel, BLU-667 had FDA breakthrough therapy designation in RET-fusion-positive NSCLC that progressed following platinum-based chemotherapy. All these data support expansion of BLU-677 and LOXO-292 in continuing enrolment of other RET-altered solid tumor groups, including BC.

Safety outcomes and preliminary antitumor activity results of RXDX-105 were evaluated in a Phase I/Ib study that has just recently been published^[112]^.

Lastly, a different approach took advantage of the finding that *RET* is also regulated by IL-6, an inflammatory cytokine which is involved in FAK-mediated control of migration and metastatic capability of ER+ BC cells^[63,113]^. A RET-IL-6 loop was identified with RET activation increasing IL-6 levels that, in turn, induces *RET* expression. Thus, RET inhibition might limit IL-6 signaling and, opposite, RET-mediated cell migration might be reduced by anti-IL-6 antibody^[63,113]^.

### Mechanisms of drug resistance in BC

Unfortunately, despite the numerous targeted therapies, many patients develop resistance after an initial promising response, whose underlying molecular mechanisms remain largely unknown.

Nevertheless, inhibition of RET may be an effective treatment option in RET-altered BC patients and, most importantly, a combined treatment may delay drug resistance, dissemination of tumor cells and metastasis.

Endocrine therapy is the main option for patients with ER±+ BC. In the last decade, new forms of endocrine therapy have been developed^[60]^ including: (1) SERMs, as tamoxifen, which binds ER and selectively inhibits or stimulates estrogen; (2) selective ER down-regulators (SERDs), or anti-estrogens, such as fulvestrant or faslodexW, blocking ER and downstream signals; and (3) AIs, inhibiting the conversion from androgens to estrogens. There are two types of AIs: steroidal, as exemestane (aromasinW), and non-steroidal, as letrozole (femaraW) and anastrazole (arimidexW). In postmenopausal women, AIs are the first-line treatment choice^[114]^.

Cross-talk between ER± and upstream kinases, with the consequent estrogen-independent activation of the Era, is one of the most-studied causes of HR^[115-117]^.

**Figure 3:**
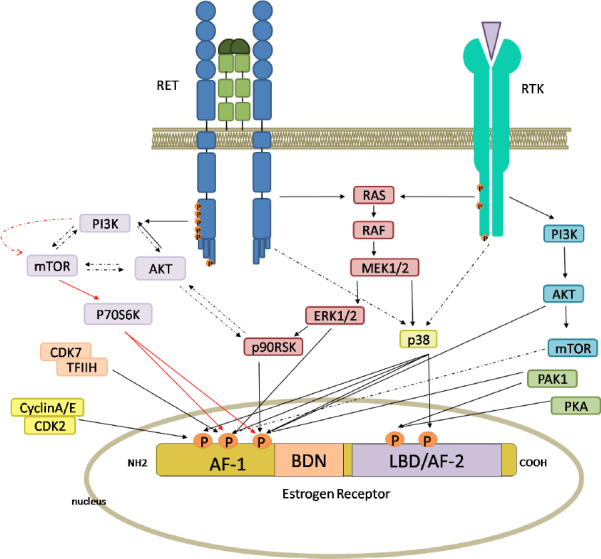
Intracellular pathways, switched on by RET and receptor tyrosine kinase (RTK), leading to the activation of estrogen receptor (ER). The activation of RET and other RTKs by specific ligands and growth factors induces both the RAS/RAF/ERK pathways and the PI3K/AKT/mTOR pathways. Phosphorylation of ER can be mediated directly by ERK, AKT, mTOR, p38, p706SK and p90RSK, or other intracellular kinases. ER has five phosphorylation sites, three in the AF-1 domain and two in the AF-2 domain. Red arrows indicate the principal RET-dependent ER-phosphorylation site. Full and broken arrows indicate known and hypothetical interactions, respectively. This figure was created by Biorender.com

Andreucci *et al*.^[109]^ (2016) demonstrated that a major cause of AI resistance is ligand-independent ER activation induced by activation of growth factor receptor(s) through PI3K/AKT/mTOR or MAPK^[64,118-121]^. Thus, BC cells are able to escape the growth-control effect of endocrine drugs by increasing estrogen-independent ER activity [Figure 3].

In particular, HER2 overexpression causes ER± phosphorylation and resistance to tamoxifen *in vitro*^[122-126]^ and is associated with HR *in vivo*^[127-129]^.

The monoclonal antibody trastuzumab (TZMB) represents the main therapeutic option for HER2+ BCs^[130]^. TZMB increased tumor response, PFS and OS in metastatic BCs. However, its efficacy is limited by cancer resistance, either *de novo* or acquired, after the first years of treatment^[131,132]^.

In recent years, many studies demonstrated that the *RET* gene and its pathway play a key role in the response to endocrine therapy in ER+ BCs by inducing ER phosphorylation, cell growth and survival^[18,60,64,65]^.

The mechanism(s) of such a resistance is far from being understood. Treatment with estrogens can lead to upregulation of RET. This latter, once activated by GDNF, results in enhanced ER phosphorylation and therefore ER downstream signaling^[60]^.

Although oncogenic *RET* mutations are not common in BC, *RET* overexpression or rearrangements in ER+ BC, both cell lines and tumors, are known^[60]^. *RET* overexpression is associated with decreased metastasis-free survival and OS in BCs^[65]^. On the other hand, RET inhibition reduced growth and metastasis^[65,85]^. Moreover, increased *RET* expression has been reported in patients who did not respond to tamoxifen, indicating a key role for RET in HR^[64]^.

Indeed, RET and ER pathways do functionally interact^[133]^, thus inducing HR by cross-talking^[18]^. Therefore, inhibition of RET might render BC cells sensitive to endocrine therapy.

Plaza-Menacho *et al*.^[64]^ (2010) showed that RET activation by GDNF in ER±+ BC cells induced both ER± phosphorylation on Ser118 and Ser167 and estrogen-independent activation of ER± activity^[64]^. They also showed a key role of mTOR in the downstream signaling pathway. In experiments with tamoxifen in MCF7 cells, RET downregulation increased sensitivity to anti-proliferative effects of the drug, while GDNF produced protective effects. In tamoxifen-resistant cells, sensitivity to tamoxifen is restored by targeting RET^[64]^.

Spanheimer *et al*.^[81]^ (2014) reported that, in a MCF7 xenograft model, RET inhibition increases the efficacy of anti-estrogen drugs. In this light, a therapy combining tamoxifen and vandetanib might be a promising therapeutic strategy for *RET*-expressing BCs.

Horibata *et al*.^[134]^ (2018) showed that, in ER+ BCs, either endocrine resistant or sensitive, RET has a functional signaling pathway. However, responsive BC cells lack any RET ligand, “which is needed to induce HR, and consistently GDNF transcription causes resistance in the ER+ MCF7 cell line. GDNF produced by resistant cells is secreted and activates the RET signaling in nearby cells. Therefore, RET ligand expression can predict the responsiveness to endocrine treatment and the clinical outcome.

In addition, the ectopic expression of ARTN induced resistance to tamoxifen and fulvestrant in MCF7 cells and in xenografts^[64]^. However, the mechanism by which ARTN is involved in downstream RET signaling still needs to be clarified.

Noteworthy, inflammatory response seems to be regulated by GDNF-RET pathways. In particular, genes associated with poor prognosis and HR were shown enriched with interferon-related genes^[18]^. Moreover, in addition to the ER-dependent activation of RET, it has been found that IL-6 is able to induce *RET* transcription^[63]^. In addition, GDNF is induced *in vitro* by TNF-± and IL-1β^[62,135,136]^, inflammatory cytokines secreted by tumor-associated macrophages (TAM) in BC. Intriguingly, in MCF7 xenografts, GDNF was related to the tumor infiltrating fibroblasts (TIF) and the invasive margin of the lesion^[62]^. A vicious loop involving RET signaling might influence cell survival and resistance to therapy in ER+ BC: the estrogen-induced upregulation of RET and ARTN promote tumorigenesis by recruiting inflammatory cells that can, on their side, induce GDNF.

Gattelli *et al*.^[65]^ (2013) showed that elevated RET levels correlate with shorter metastasis-free survival and that RET activation induces pro-inflammatory cytokines during endocrine treatment, confirming RET as a novel druggable target. They also showed that fulvestrant-induced IL-6 production enhances *RET* expression, thus demonstrating a RET-IL-6 expression loop^[65]^.

These findings, along with the upregulation of GFR±3 in endocrine resistant models^[126]^, implicates that increased RET activation in endocrine-resistant BCs may promote tumor growth either through ER-dependent ER phosphorylation increase or via an ER-independent mechanism activating MAPKs and/or inducing pro-survival genes such as BCL-2.

Finally, it has recently been shown that RET activation may be relevant in TNBCs^[137]^ and HER2+ BCs^[138]^, being low RET levels also found in ER±- and TNBC tumors. TFAP2C has been shown to induce ER± independent *RET* expression in MDA-MB-453 cells, with important implications for the ER±- BCs^[84]^.

### How RET inhibitors might overcome drug resistance

Overall, since RET level and activation of its kinase are linked to HR, a solid biological rationale for combining endocrine drugs with RET inhibitors exists.

Since administration of letrozole with a RET inhibitor has demonstrated improved efficacy over letrozole alone in preclinical models, an increasing number of clinical trials has started to evaluate the use of RET inhibitors to enhance sensitivity and to reduce HR in BCs (clinicaltrials.gov).

Both RET and ER± are strictly connected in the cell proliferation and survival control in BC, thus suggesting the combined targeting of both pathways^[83]^. Indeed, RET inhibition increases the efficacy of antiestrogen drugs, and the tamoxifen + vandetanib treatment has been proved as a promising approach for RET+ BCs^[81]^.

Spanheimer *et al*.^[81,84]^ reported that tamoxifen and vandetanib have similar efficacy in limiting MCF7 tumor growth *in vivo*^[81,84]^. On the other hand, the combination of these two drugs was much more efficient than either drug alone^[139]^. However, Gattelli *et al*.^[65]^ also studied the combination of RET inhibitor with hormonal therapy in a BC murine model and obtained different results.

Griseri *et al*.^[33]^ (2016) compared the MCF7 and T47D cell lines to disclose the molecular mechanisms able to account for their different RET levels. In particular, the MCF7 and T47D BC cell lines were characterized for the two candidate *RET* variants that had discordant genotype (rs12247456:AA *vs.* GG; rs2435357:CC *vs* TT). These data are in agreement with the observation that T47D, expressing lower *RET* mRNA level, are homozygous for the T allele of rs2435357, a genotype known to associate with RET downregulation^[37,140]^. To verify the effect of rs2435357C>T SNP *in vivo*, 93 ER±+ BC patients were genotyped. Consistent with the observation that RET overexpression leads to poor prognosis in ER±+ BC, the presence of at least one variant allele (CT or TT) was associated with a longer OS when compared to patients carrying the wt CC alleles, thus suggesting that the *RET*+3 SNP represent a reliable prognostic factor in these patients.

Moreover, Hatem *et al*.^[90]^ (2016) reported the potential of vandetanib in the treatment of chemotherapy for TZMB resistant ER- BCs. In RET or EGFR expressing models, vandetanib showed a remarkable tumor regression, an effect ascribed to inhibition of RET or EGFR phosphorylation and downstream signaling pathways. Subsets of patients expressing RET, such as < 10% of TNBCs and 20%-40% of HER2+ BCs, as well as those expressing EGFR, approximately 6%, might have benefited from treatments with vandetanib.

Finally, given the RET expression in the peripheral nervous system in adults, we need to be aware that down-regulating drugs might lead to peripheral neuropathy^[107]^. Therefore, RET inhibitors with potential clinical application in BC and reduced toxicity should be developed.

In summary, the involvement of RET in the pathogenesis of BC and in the development of ER±+ tumors is confirmed by several independent studies and a strong body of evidence confirms that RET might be an effective target to enhance sensitivity of BC to antitumoral therapy and to overcome drug resistance.

## Conclusion

Improved knowledge around BC and persisting unsolved aspects of its biology, does suggest we keep on current research strategies: (1) patient stratification, according to gene expression patterns^[141]^, distinct response to treatments, recurrence and survival^[87,142,143]^, will result useful to search for further suitable markers; (2) around 40%-50% of BC patients develop endocrine-resistant BC^[144]^, thus disclosing the mechanism of HR has become a priority in reducing the BC mortality; (3) RTKs have emerged as promising therapeutic targets to modulate the response to therapy in BCs, mostly mediated by their amplification or overexpression. Unfortunately, thus far, there is no evidence for the direct involvement of amplification or overexpression of RTK in ER+ disease, a circumstance explaining why no RTKi has been approved yet; (4) *RET* has emerged as driving oncogenesis not only in thyroid tumors but also in lung cancers as well as in other epithelial tumors (e.g., ERα+ BC)^[79]^. The development of new biomarkers and drugs will require a better understanding of RET-mediated signaling pathways and their crosstalk with ERα signaling; (5) inhibitors actually found to also hit RET in screenings designed to target other RTKs have revealed the emerging role of RET as a potential druggable target. Nevertheless, no RET-specific inhibitor has been developed thus far; and (6) as downstream RET pathways modulating ER activity are shared with other RTKs, combining endocrine therapies with inhibitors targeting shared signaling components has been proposed as a promising approach in ER- and RTK signaling-positive patients^[145]^. Indeed, combination approaches will allow larger subsets of patients to become eligible for trials, besides preventing secondary resistance in highly mutable tumors.

## Declarations

### Copyright

© The Author(s) 2019.

## References

[1] Jemal A, Bray F, Center MM, Ferlay J, Ward E (2011). Global cancer statistics.. CA Cancer J Clin.

[2] Tao Z, Shi A, Lu C, Song T, Zhang Z (2015). Breast cancer: epidemiology and etiology.. Cell Biochem Biophys.

[3] Anastasiadi Z, Lianos GD, Ignatiadou E, Harissis HV, Mitsis M (2017). Breast cancer in young women: an overview.. Updates Surg.

[4] van der Groep P, van der Wall E, van Diest PJ (2011). Pathology of hereditary breast cancer.. Cell Oncol (Dordr).

[5] Shiovitz S, Korde LA (2015). Genetics of breast cancer: a topic in evolution.. Ann Oncol.

[6] Watson IR, Takahashi K, Futreal PA, Chin L (2013). Emerging patterns of somatic mutations in cancer.. Nat Rev Genet.

[7] Bertheau P, Lehmann-Che J, Varna M, Dumay A, Poirot B (2013). p53 in breast cancer subtypes and new insights into response to chemotherapy.. Breast..

[8] Goncalves R, Warner WA, Luo J, Ellis MJ (2014). New concepts in breast cancer genomics and genetics.. Breast Cancer Res.

[9] Byler S, Goldgar S, Heerboth S, Leary M, Housman G (2014). Genetic and epigenetic aspects of breast cancer progression and therapy.. Anticancer Res.

[10] Spanheimer PM, Lorenzen AW, De Andrade JP, Kulak MV, Carr JC (2015). Receptor tyrosine kinase expression predicts response to sunitinib in breast cancer.. Ann Surg Oncol.

[11] Sorlie T, Perou CM, Tibshirani R, Aas T, Geisler S (2001). Gene expression patterns of breast carcinomas distinguish tumor subclasses with clinical implications.. Proc Natl Acad Sci USA.

[12] Spanheimer PM, Carr JC, Thomas A, Sugg SL, Scott-Conner CE (2013). The response to neoadjuvant chemotherapy predicts clinical outcome and increases breast conservation in advanced breast cancer.. Am J Surg.

[13] Rouzier R, Perou CM, Symmans WF, Ibrahim N, Cristofanilli M (2005). Breast cancer molecular subtypes respond differently to preoperative chemotherapy.. Clin Cancer Res.

[14] Meyers MO, Klauber-Demore N, Ollila DW, Amos KD, Moore DT (2011). Impact of breast cancer molecular subtypes on locoregional recurrence in patients treated with neoadjuvant chemotherapy for locally advanced breast cancer.. Ann Surg Oncol.

[15] Livi L, Palar F, Saieva C, Simontacchi G, Nori J (2006). Breast cancer in the elderly: treatment of 1500 patients.. Breast J.

[16] Huang B, Warner M, Gustafsson JA (2015). Estrogen receptors in breast carcinogenesis and endocrine therapy.. Mol Cell Endocrinol.

[17] Brufsky AM (2014). Predictive and prognostic value of the 21-gene recurrence score in hormone receptor-positive, node-positive breast cancer.. Am J Clin Oncol.

[18] Morandi A, Martin LA, Gao Q, Pancholi S, Mackay A (2013). GDNF-RET signaling in ER-positive breast cancers is a key determinant of response and resistance to aromatase inhibitors.. Cancer Res.

[19] Forbes JF, Cuzick J, Buzdar A, Howell A, Tobias JS (2008). Effect of anastrozole and tamoxifenas adjuvant treatment for early-stage breast cancer: 100-month analysis of the ATAC trial.. Lancet Oncol.

[20] Winer EP, Hudis C, Burstein HJ, Wolff AC, Pritchard KI (2005). American society of clinical oncology technology assessment on the use of aromatase inhibitors as adjuvant therapy for postmenopausal women with hormone receptor-positive breast cancer: status report 2004.. J Clin Oncol.

[21] Bousquet G, Varna M, Ferreira I, Wang L, Mongiat-Artus P (2013). Differential regulation of sunitinib targets predicts its tumor-type-specific effect on endothelial and/or tumor cell apoptosis.. Cancer Chemother Pharmacol.

[22] Aparicio-Gallego G, Blanco M, Figueroa A, García-Campelo R, Valladares-Ayerbes M (2011). New insights into molecular mechanisms of sunitinib-associated side effects.. Mol Cancer Ther.

[23] Kudo M (2010). Current status of molecularly targeted therapy for hepatocellular carcinoma: clinical practice.. Int J Clin Oncol.

[24] Lei JT, Gou X, Seker S, Ellis MJ (2019). ESR1 alterations and metastasis in estrogen receptor positive breast cancer.. J Cancer Metastasis Treat.

[25] Arpino G, Wiechmann L, Osborne CK, Schiff R (2008). Crosstalk between the estrogen receptor and the HER tyrosine kinase receptor family: molecular mechanism and clinical implications for endocrine therapy resistance.. Endocr Rev.

[26] Martin LA, Farmer I, Johnston SR, Ali S, Marshall C (2003). Enhanced estrogen receptor (ER) alpha, ERBB2, and MAPK signal transduction pathways operate during the adaptation of MCF-7 cells to long term estrogen deprivation.. J Biol Chem.

[27] Stephen RL, Shaw LE, Larsen C, Corcoran D, Darbre PD (2001). Insulin-like growth factor receptor levels are regulated by cell density and by long term estrogen deprivation in MCF7 human breast cancer cells.. J Biol Chem.

[28] Miller TW, Hennessy BT, Gonzalez-Angulo AM, Fox EM, Mills GB (2010). Hyperactivation of phosphatidylinositol-3 kinase promotes escape from hormone dependence in estrogen receptor-positive human breast cancer.. J Clin Invest.

[29] Nayar U, Cohen O, Kapstad C, Cuoco MS, Waks AG (2019). Acquired HER2 mutations in ER+ metastatic breast cancer confer resistance to estrogen receptor-directed therapies.. Nat Genet.

[30] Zabransky DJ, Park BH (2014). Estrogen receptor and receptor tyrosine kinase signaling: use of combinatorial hormone and epidermal growth factor receptor/human epidermal growth factor receptor 2-targeted therapies for breast cancer.. J Clin Oncol.

[31] Plaza-Menacho I, Mologni L, Sala E, Gambacorti-Passerini C, Magee AI (2007). Sorafenib functions to potently suppress RET tyrosine kinase activity by direct enzymatic inhibition and promoting RET lysosomal degradation independent of proteasomal targeting.. J Biol Chem.

[32] Gschwind A, Fischer OM, Ullrich A (2004). The discovery of receptor tyrosine kinases: targets for cancer therapy.. Nat Rev Cancer.

[33] Griseri P, Garrone O, Lo Sardo A, Monteverde M, Rusmini M (2016). Genetic and epigenetic factors affect RET gene expression in breast cancer cell lines and influence survival in patients.. Oncotarget.

[34] Airaksinen MS, Titievsky A, Saarma M (1999). GDNF family neurotrophic factor signaling: four masters, one servant?. Mol Cell Neurosci.

[35] Arighi E, Borrello MG, Sariola H (2005). RET tyrosine kinase signaling in development and cancer.. Cytokine Growth Factor Rev.

[36] Mulligan LM (2014). RET revisited: expanding the oncogenic portfolio.. Nat Rev Cancer.

[37] Raue F, Frank-Raue K (2018). update on multiple endocrine neoplasia type 2: focus on medullary thyroid carcinoma.. J Endocr Soc.

[38] Dionigi G, Bianchi V, Rovera F, Boni L, Piantanida E (2007). Medullary thyroid carcinoma: surgical treatment advances.. Expert Rev Anticancer Ther.

[39] Wells SA Jr, Pacini F, Robinson BG, Santoro M (2013). Multiple endocrine neoplasia type 2 and familial medullary thyroid carcinoma: an update.. J Clin Endocrinol Metab.

[40] Toledo SPA, Cortina MA, Toledo RA, Lourenço DM (2006). Impact of RET proto-oncogene analysis on the clinical management of multiple endocrine neoplasia type 2.. Clinics.

[41] Moura MM, Cavaco BM, Pinto AE, Domingues R, Santos JR (2009). Correlation of RET somatic mutations with clinicopathological features in sporadic medullary thyroid carcinomas.. Br J Cancer.

[42] Dvorakova S, Vaclavikova E, Sykorova V, Vcelak J, Novak Z (2008). Somatic mutations in the RET proto-oncogene in sporadic medullary thyroid carcinomas.. Mol Cell Endocrinol.

[43] Elisei R, Cosci B, Romei C, Bottici V, Renzini G (2008). Prognostic significance of somatic RET oncogene mutations in sporadic medullary thyroid cancer: a 10-year follow-up study.. J Clin Endocrinol Metab.

[44] Kurzrock R, Sherman SI, Ball DW, Forastiere AA, Cohen RB (2011). Activity of XL184 (cabozantinib), an oral tyrosine kinase inhibitor, in patients with medullary thyroid cancer.. J Clin Oncol.

[45] Santoro M, Melillo RM, Fusco A (2006). RET/PTC activation in papillary thyroid carcinoma: European Journal of Endocrinology Prize Lecture.. Eur J Endocrinol.

[46] Ciampi R, Nikiforov YE (2007). RET/PTC rearrangements and BRAF mutations in thyroid tumorigenesis.. Endocrinology.

[47] Subbiah V, Gainor JF, Rahal R, Brubaker JD, Kim JL (2018). Precision targeted therapy with BLU-667 for RET-driven cancers.. Cancer Discov.

[48] Kato S, Subbiah V, Marchlik E, Elkin SK, Carter JL (2017). RET aberrations in diverse cancers: next-generation sequencing of 4,871 patients.. Clin Cancer Res.

[49] Ballerini P, Struski S, Cresson C, Prade N, Toujani S (2012). RET fusion genes are associated with chronic myelomonocytic leukemia and enhance monocytic differentiation.. Leukemia.

[50] Gozgit JM, Chen TH, Song Y, Wardwell S, Wang F (2018). RET fusions observed in lung and colorectal cancers are sensitive to ponatinib.. Oncotarget.

[51] Mulligan LM (2019). GDNF and the RET receptor in cancer: new insights and therapeutic potential.. Front Physiol.

[52] Skálová A, Ptáková N, Santana T, Agaimy A, Ihrler S (2019). NCOA4-RET and TRIM27-RET are characteristic gene fusions in salivary intraductal carcinoma, including invasive and metastatic tumors: is “Intraductal” correct?. Am J Surg Pathol.

[53] Wiesner T, He J, Yelensky R, Esteve-Puig R, Botton T (2014). Kinase fusions are frequent in Spitz tumours and spitzoid melanomas.. Nat Commun.

[54] Paratala BS, Chung JH, Williams CB, Yilmazel B, Petrosky W (2018). RET rearrangements are actionable alterations in breast cancer.. Nat Commun.

[55] Griseri P, Bachetti T, Puppo F, Lantieri F, Ravazzolo R (2005). A common haplotype at the 5’ end of the RET proto-oncogene, overrepresented in Hirschsprung patients, is associated with reduced gene expression.. Hum Mutat.

[56] Emison ES, McCallion AS, Kashuk CS, Bush RT, Grice E (2005). A common sex-dependent mutation in a RET enhancer underlies Hirschsprung disease risk.. Nature.

[57] Lantieri F, Griseri P, Puppo F, Campus R, Martucciello G (2006). Haplotypes of the human RET proto-oncogene associated with Hirschsprung disease in the Italian population derive from a single ancestral combination of alleles.. Ann Hum Genet.

[58] Borun P, Jerzy S, Ziemnicka K, Kubaszewski L, Lipinski D (2012). Absence of the RET+3:T allele in the MTC patients.. Hered Cancer Clin Pract.

[59] Emison ES, Garcia-Barcelo M, Grice EA, Lantieri F, Amiel J (2010). Differential contributions of rare and common, coding and noncoding Ret mutations to multifactorial Hirschsprung disease liability.. Am J Hum Genet.

[60] Morandi A, Plaza-Menacho I, Isacke CM (2011). RET in breast cancer: functional and therapeutic implications.. Trends Mol Med.

[61] Tozlu S, Girault I, Vacher S, Vendrell J, Andrieu C (2006). Identification of novel genes that co-cluster with estrogen receptor alpha in breast tumor biopsy specimens, using a large-scale real-time reverse transcription-PCR approach.. Endocr Relat Cancer.

[62] Esseghir S, Todd SK, Hunt T, Poulsom R, Plaza-Menacho I (2007). A role for glial cell derived neurotrophic factor induced expression by inflammatory cytokines and RET/GFR alpha 1 receptor up-regulation in breast cancer.. Cancer Res.

[63] Boulay A, Breuleux M, Stephan C, Fux C, Brisken C (2008). The Ret receptor tyrosine kinase pathway functionally interacts with the ERalpha pathway in breast cancer.. Cancer Res.

[64] Plaza-Menacho I, Morandi A, Robertson D, Pancholi S, Drury S (2010). Targeting the receptor tyrosine kinase RET sensitizes breast cancer cells to tamoxifen treatment and reveals a role for RET in endocrine resistance.. Oncogene.

[65] Gattelli A, Nalvarte I, Boulay A, Roloff TC, Schreiber M (2013). Ret inhibition decreases growth and metastatic potential of estrogen receptor positive breast cancer cells.. EMBO Mol Med.

[66] Nguyen M, Miyakawa S, Kato J, Mori T, Arai T (2015). Preclinical efficacy and safety assessment of an antibody-drug conjugate targeting the c-RET proto-oncogene for breast carcinoma.. Clin Cancer Res.

[67] Drosten M, Putzer BM (2006). Mechanisms of disease: cancer targeting and the impact of oncogenic RET for medullary thyroid carcinoma therapy.. Nat Clin Pract Oncol.

[68] Plaza-Menacho I, Burzynski GM, de Groot JW, Eggen BJ, Hofstra RM (2006). Current concepts in RET-related genetics, signaling and therapeutics.. Trends Genet.

[69] Lanzi C, Cassinelli G, Nicolini V, Zunino F (2009). Targeting RET for thyroid cancer therapy.. Biochem Pharmacol.

[70] Scott RP, Eketjäll S, Aineskog H, Ibáñez CF (2005). Distinct turnover of alternatively spliced isoforms of the RET kinase receptor mediated by differential recruitment of the Cbl ubiquitin ligase.. J Biol Chem.

[71] Hickey JG, Myers SM, Tian X, Zhu SJ, V Shaw JL (2009). RET-mediated gene expression pattern is affected by isoform but not oncogenic mutation.. Genes Chromosomes Cancer.

[72] Le Hir H, Charlet-Berguerand N, de Franciscis V, Thermes C (2002). 5’-End RET splicing: absence of variants in normal tissues and intron retention in pheochromocytomas.. Oncology.

[73] Ferlay J, Soerjomataram I, Dikshit R, Eser S, Mathers C (2015). Cancer incidence and mortality worldwide: sources, methods and major patterns in GLOBOCAN 2012.. Int J Cancer.

[74] Yuan ZL, Guan YJ, Wang L, Wei W, Kane AB (2004). Central role of the threonine residue within the p+1 loop of receptor tyrosine kinase in STAT3 constitutive phosphorylation in metastatic cancer cells.. Mol Cell Biol.

[75] Borrello MG, Ardini E, Locati LD, Greco A, Licitra L (2013). RET inhibition: implications in cancer therapy.. Expert Opin Ther Targets.

[76] de Groot JW, Plaza Menacho I, Schepers H, Drenth-Diephuis LJ, Osinga J (2006). Cellular effects of imatinib on medullary thyroid cancer cells harboring multiple endocrine neoplasia type 2A and 2B associated RET mutations.. Surgery.

[77] Pacifico F, Crescenzi E, Mellone S, Iannetti A, Porrino N (2010). Nuclear factor-{kappa}B contributes to anaplastic thyroid carcinomas through up-regulation of miR-146a.. J Clin Endocrinol Metab.

[78] Castellone MD, De Falco V, Rao DM, Bellelli R, Muthu M (2009). The beta-catenin axis integrates multiple signals downstream from RET/papillary thyroid carcinoma leading to cell proliferation.. Cancer Res.

[79] Plaza-Menacho I, Barnouin K, Barry R, Borg A, Orme M (2016). RET functions as a dual-specificity kinase that requires allosteric inputs from juxtamembrane elements.. Cell Rep.

[80] Uhlen M, Zhang C, Lee S, Sjöstedt E, Fagerberg L (2017). A pathology atlas of the human cancer transcriptome.. Science.

[81] Spanheimer PM, Park JM, Askeland RW, Kulak MV, Woodfield GW (2014). Inhibition of RET increases the efficacy of anti-estrogen and is a novel treatment strategy for luminal breast cancer.. Clin Cancer Res.

[82] Sommer S, Fuqua SA (2001). Estrogen receptor and breast cancer.. Semin Cancer Biol.

[83] Stine ZE, McGaughey DM, Bessling SL, Li S, McCallion AS (2011). Steroid hormone modulation of RET through two estrogen responsive enhancers in breast cancer.. Hum Mol Genet.

[84] Spanheimer PM, Woodfield GW, Cyr AR, Kulak MV, White-Baer LS (2013). Expression of the RET proto-oncogene is regulated by TFAP2C in breast cancer independent of the estrogen receptor.. Ann Surg Oncol.

[85] Wang C, Mayer JA, Mazumdar A, Brown PH (2012). The rearranged during transfection/papillary thyroid carcinoma tyrosine kinase is an estrogen dependent gene required for the growth of estrogen receptor positive breast cancer cells.. Breast Cancer Res Treat.

[86] Tan SK, Lin ZH, Chang CW, Varang V, Chng KR (2011). AP-2 regulates oestrogen receptor-mediated long-range chromatin interaction and gene transcription.. EMBO J.

[87] Williams T, Tjian R (1991). Analysis of the DNA-binding and activation properties of the human transcription factor AP-2.. Genes Dev.

[88] McPherson LA, Weigel RJ (1999). AP2alpha and AP2gamma: a comparison of binding site specificity and trans-activation of the estrogen receptor promoter and single site promoter constructs.. Nucleic Acids Res.

[89] De Andrade JP, Park JM, Gu VW, Woodfield GW, Kulak MV (2016). EGFR is regulated by TFAP2C in luminal breast cancer and is a target for Vandetanib.. Mol Cancer Ther.

[90] Hatem R, Labiod D, Château-Joubert S, de Plater L, El Botty R (2016). Vandetanib as a potential new treatment for estrogen receptor-negative breast cancers.. Int J Cancer.

[91] de Groot JW, Zonnenberg BA, van Ufford-Mannesse PQ, de Vries MM, Links TP (2007). A phase II trial of imatinib therapy for metastatic medullary thyroid carcinoma.. J Clin Endocrinol Metab.

[92] Phay JE, Shah MH (2010). Targeting RET receptor tyrosine kinase activation in cancer.. Clin Cancer Res.

[93] Polverino A, Coxon A, Starnes C, Diaz Z, DeMelfi T (2006). AMG 706, an oral, multikinase inhibitor that selectively targets vascular endothelial growth factor, platelet-derived growth factor, and kit receptors, potently inhibits angiogenesis and induces regression in tumor xenografts.. Cancer Res.

[94] Caenepeel S, Renshaw-Gegg L, Baher A, Bush TL, Baron W (2010). Motesanib inhibits Kit mutations associated with gastrointestinal stromal tumors.. J Exp Clin Cancer Res.

[95] McPherson LA, Weigel RJ (1999). AP2alpha and AP2gamma: a comparison of binding site specificity and trans-activation of the estrogen receptor promoter and single site promoter constructs.. Nucleic Acids Res.

[96] Plaza-Menacho I, Morandi A, Mologni L, Boender P, Gambacorti-Passerini C (2011). Focal adhesion kinase (FAK) binds RET kinase via its FERM domain, priming a direct and reciprocal RET-FAK transactivation mechanism.. J Biol Chem.

[97] Mulligan LM (2014). RET revisited: expanding the oncogenic portfolio.. Nat Rev Cancer.

[98] Spanheimer PM, Cyr AR, Gillum MP, Woodfield GW, Askeland RW (2014b). Distinct pathways regulated by RET and estrogen receptor in luminal breast cancer demonstrate the biological basis for combination therapy.. Ann Surg.

[99] Li L, Yu J, Jiao S, Wang W, Zhang F (2018). Vandetanib (ZD6474) induces antiangiogenesis through mTOR-HIF-1 alpha-VEGF signaling axis in breast cancer cells.. Onco Targets Ther.

[100] Zhang M, Gao CE, Chen WL, Tang YY, Nie JY (2018). Opposite response to hypoxia by breast cancer cells between cell proliferation and cell migration: a clue from microRNA expression profile.. Oncol Lett.

[101] Kapinova A, Kubatka P, Zubor P, Golubnitschaja O, Dankova Z (2018). The hypoxia-responsive long non-coding RNAs may impact on the tumor biology and subsequent man¬agement of breast cancer.. Biomed Pharmacother.

[102] Wilhelm SM, Carter C, Tang L, Wilkie D, McNabola A (2004). BAY 43-9006 exhibits broad spectrum oral antitumor activity and targets the RAF/MEK/ERK pathway and receptor tyrosine kinases involved in tumor progression and angiogenesis.. Cancer Res.

[103] Carlomagno F, Anaganti S, Guida T, Salvatore G, Troncone G (2006). BAY 43-9006 inhibition of oncogenic RET mutants.. J Natl Cancer Inst.

[104] Kim DW, Jo YS, Jung HS, Chung HK, Song JH (2006). An orally administered multitarget tyrosine kinase inhibitor, SU11248, is a novel potent inhibitor of thyroid oncogenic RET/papillary thyroid cancer kinases.. J Clin Endocrinol Metab.

[105] Karaman MW, Herrgard S, Treiber DK, Gallant P, Atteridge CE (2008). A quantitative analysis of kinase inhibitor selectivity.. Nat Biotechnol.

[106] Wedge SR, Ogilvie DJ, Dukes M, Kendrew J, Chester R (2002). ZD6474 inhibits vascular endothelial growth factor signaling, angiogenesis, and tumor growth following oral administration.. Cancer Res.

[107] Wells SA Jr, Gosnell JE, Gagel RF, Moley J, Pfister D (2010). Vandetanib for the treatment of patients with locally advanced or metastatic hereditary medullary thyroid cancer.. J Clin Oncol.

[108] Herbst RS, Heymach JV, O’Reilly MS, Onn A, Ryan AJ (2007). Vandetanib (ZD6474): an orally available receptor tyrosine kinase inhibitor that selectively targets pathways critical for tumor growth and angiogenesis.. Expert Opin Investig Drugs.

[109] Andreucci E, Francica P, Fearns A, Martin LA, Chiarugi P (2016). Targeting the receptor tyrosine kinase RET in combination with aromatase inhibitors in ER positive breast cancer xenografts.. Oncotarget.

[110] Ackermann CJ, Stock G, Tay R, Dawod M, Gomes F (2019). Targeted therapy for RET-rearranged non-small cell lung cancer: clinical development and future directions.. Onco Targets Ther.

[111] Iams WT, Lovly CM (2018). Stop fRETting the target: next-generation RET inhibitors have arrived.. Cancer Discov.

[112] Drilon A, Fu S, Patel MR, Fakih M, Wang D (2019). A phase I/Ib trial of the VEGFR sparing multikinase RET inhibitor RXDX-105.. Cancer Discov.

[113] Morandi A, Isacke CM (2014). Targeting RET-interleukin-6 crosstalk to impair metastatic dissemination in breast cancer.. Breast Cancer Res.

[114] Forbes JF, Cuzick J, Buzdar A, Howell A, Tobias JS (2008). Effect of anastrozole and tamoxifen as adjuvant treatment for early-stage breast cancer: 100-month analysis of the ATAC trial.. Lancet Oncol.

[115] Ali S, Coombes RC (2002). Endocrine-responsive breast cancer and strategies for combating resistance.. Nat Rev Cancer.

[116] Massarweh S, Schiff R (2006). Resistance to endocrine therapy in breast cancer: exploiting estrogen receptor/growth factor signaling crosstalk.. Endocr Relat Cancer..

[117] Musgrove EA, Sutherland RL (2009). Biological determinants of endocrine resistance in breast cancer.. Nat Rev Cancer.

[118] Martin LA, Farmer I, Johnston SR, Ali S, Marshall C (2003). Enhanced estrogen receptor (ER) alpha, ERBB2, and MAPK signal transduction pathways operate during the adaptation of MCF-7 cells to long term estrogen deprivation.. J Biol Chem.

[119] Martin LA, Pancholi S, Chan CM, Farmer I, Kimberley C (2005). The anti-oestrogen ICI 182,780, but not tamoxifen, inhibits the growth of MCF-7 breast cancer cells refractory to long-term oestrogen deprivation through down-regulation of oestrogen receptor and IGF signalling.. Endocr Relat Cancer.

[120] Musgrove EA, Sutherland RL (2009). Biological determinants of endocrine resistance in breast cancer.. Nat Rev Cancer.

[121] Ma CX, Reinert T, Chmielewska I, Ellis MJ (2015). Mechanisms of aromatase inhibitor resistance.. Nat Rev Cancer.

[122] Chung YL, Sheu ML, Yang SC, Lin CH, Yen SH (2002). Resistance to tamoxifen-induced apoptosis is associated with direct interaction between Her2/neu and cell membrane estrogen receptor in breast cancer.. Int J Cancer.

[123] Osborne CK, Shou J, Massarweh S, Schiff R (2005). Crosstalk between estrogen receptor and growth factor receptor pathways as a cause for endocrine therapy resistance in breast cancer.. Clin Cancer Res.

[124] Shou J, Massarweh S, Osborne CK, Wakeling AE, Ali S (2004). Mechanisms of tamoxifen resistance: increased estrogen receptor-HER2/neu cross-talk in ER/HER2-positive breast cancer.. J Natl Cancer Inst.

[125] Shou J, Massarweh S, Osborne CK, Wakeling AE, Ali S (2004). Mechanisms of tamoxifen resistance: increased estrogen receptor-HER2/neu cross-talk in ER/HER2-positive breast cancer.. J Natl Cancer Inst.

[126] Pancholi S, Lykkesfeldt AE, Hilmi C, Banerjee S, Leary A (2008). ERBB2 influences the subcellular localization of the estrogen receptor in tamoxifen-resistant MCF-7 cells leading to the activation of AKT and RPS6KA2.. Endocr Relat Cancer.

[127] Benz CC, Scott GK, Sarup JC, Johnson RM, Tripathy D (1992). Estrogen-dependent, tamoxifen-resistant tumorigenic growth of MCF-7 cells transfected with HER2/neu.. Breast Cancer Res Treat.

[128] Yamashita H (2008t). Current research topics in endocrine therapy for breast cancer.. Int J Clin Oncol.

[129] Kok M, Holm-Wigerup C, Hauptmann M, Michalides R, Stål O (2009). Estrogen receptor-alpha phosphorylation at serine-118 and tamoxifen response in breast cancer.. J Natl Cancer Inst.

[130] Gardaneh M, Shojaei S, Kaviani A, Behnam B (2017). GDNF induces RET-SRC-HER2-dependent growth in trastuzumab-sensitive but SRC-independent growth in resistant breast tumor cells.. Breast Cancer Res Treat.

[131] Slamon DJ, Leyland-Jones B, Shak S, Fuchs H, Paton V (2001). Use of chemotherapy plus a monoclonal antibody against HER2 for metastatic breast cancer that overexpresses HER2.. N Engl J Med.

[132] Marty M, Cognetti F, Maraninchi D, Snyder R, Mauriac L (2005). Randomized phase II trial of the efficacy and safety of trastuzumab combined with docetaxel in patients with human epidermal growth factor receptor 2-positive metastatic breast cancer administered as first-line treatment: the M77001 study group.. J Clin Oncol.

[133] Hurtado A, Holmes KA, Geistlinger TR, Hutcheson IR, Nicholson RI (2008). Regulation of ERBB2 by oestrogen receptor-PAX2 determines response to tamoxifen.. Nature.

[134] Horibata S, Rice EJ, Mukai C, Marks BA, Sams K (2018). ER-positive breast cancer cells are poised for RET-mediated endocrine resistance.. PLoS One.

[135] Kuno R, Yoshida Y, Nitta A, Nabeshima T, Wang J (2006). The role of TNF-alpha and its receptors in the production of NGF and GDNF by astrocytes.. Brain Res.

[136] O’Reilly KE, Rojo F, She QB, Solit D, Mills GB (2006). mTOR inhibition induces upstream receptor tyrosine kinase signaling and activates Akt.. Cancer Res.

[137] Sariola H, Saarma M (2003). Novel functions and signalling pathways for GDNF.. J Cell Sci.

[138] Drosten M, Putzer BM (2006). Mechanisms of disease: cancer targeting and the impact of oncogenic RET for medulary thyroid carcinoma therapy.. Nat Clin Pract Oncol.

[139] Hickey JG, Myers SM, Tian X, Zhu SJ, Shaw JL (2009). RET-mediated gene expression pattern is affected by isoform but not oncogenic mutation.. Genes Chromosomes Cancer.

[140] Emison ES, Garcia-Barcelo M, Grice EA, Lantieri F, Amiel J (2010). Differential contributions of rare and common, coding and noncoding Ret mutations to multifactorial Hirschsprung disease liability.. Am J Hum Genet.

[141] Sorlie T, Perou CM, Tibshirani R, Aas T, Geisler S (2001). Gene expression patterns of breast carcinomas distinguish tumor subclasses with clinical implications.. Proc Natl Acad Sci U S A.

[142] Williams T, Tjian R (1991). Analysis of the DNA-binding and activation properties of the human transcription factor AP-2.. Genes Dev.

[143] Woodfield GW, Chen Y, Bair TB, Domann FE, Weigel RJ (2010). Identification of primary gene targets of TFAP2C in hormone responsive breast carcinoma cells.. Genes Chromosomes Cancer.

[144] Ma CX, Sanchez CG, Ellis MJ (2009). Predicting endocrine therapy responsiveness in breast cancer.. Oncology (Williston Park).

[145] Rosen LS, Ashurst HL, Chap L (2010). Targeting signal transduction pathways in metastatic breast cancer: a comprehensive review.. Oncologist.

